# Multi-frame acquisition scheme for efficient energy-dispersive X-ray magnetic circular dichroism in pulsed high magnetic fields at the Fe *K*-edge

**DOI:** 10.1107/S090904951100080X

**Published:** 2011-01-29

**Authors:** Cornelius Strohm, Florian Perrin, Marie-Christine Dominguez, Jon Headspith, Peter van der Linden, Olivier Mathon

**Affiliations:** a European Synchrotron Radiation Facility, 6 rue Jules Horowitz, 38043 Grenoble, France; b Science and Technology Facilities Council, Daresbury Laboratory, Warrington WA4 4AD, UK

**Keywords:** energy-dispersive XMCD, high magnetic field, detection

## Abstract

Using a fast silicon strip detector, a multi-frame acquisition scheme was implemented to perform energy-dispersive X-ray magnetic circular dichroism at the iron *K*-edge in pulsed high magnetic fields. The acquisition scheme makes use of the entire field pulse. The quality of the signal obtained from samples of ferrimagnetic erbium iron garnet allows for quantitative evaluation of the signal amplitude. Below the compensation point, two successive field-induced phase transitions and the reversal of the net magnetization of the iron sublattices in the intermediate phase were observed.

## Abstract 2

A multi-frame acquisition scheme for efficient energy-dispersive X-ray magnetic circular dichroism at the Fe *K*-edge is presented.

## 1. Introduction

The development of X-ray magnetic circular dichroism (XMCD) in pulsed magnetic fields (PMF) (Mathon *et al.*, 2007 ▶) has significantly increased the field range accessible for XMCD studies from the limits of current superconducting magnets to fields of 30 T and beyond. The elemental and orbital selectivity of XMCD make it an important tool for studying the microscopic physics of magnetism and magnetic phase transitions at high fields. Recent examples of scientific applications include the study of magnetic-field-induced valence transitions (Matsuda *et al.*, 2009 ▶) and magneto-structural effects (Sikora *et al.*, 2009 ▶).

Two different acquisition schemes are currently used to perform XMCD studies in PMF: in energy-scanning XMCD a series of field pulses is performed at each energy, using fast photodiodes and amplifiers capable of following the magnetic field pulse to detect the signal. This provides excellent consistency of the magnetic field dependence, while the energy spectra are constructed point by point. In energy-dispersive (ED) XMCD a focusing polychromator is used to introduce a correlation between the propagation direction and the photon energy. Using a position-sensitive detector the XMCD spectra are acquired simultaneously, resulting in excellent quality and resolution of the energy spectra. Up to now, however, ED-XMCD in PMF has been limited to the acquisition of one detector frame around peak field.

The present paper describes a multi-frame acquisition scheme for ED-XMCD. A series of spectra is acquired during the field pulse, thus combining the high quality of the energy spectra usually obtained in ED-XMCD with the consistency of the field dependence achieved in energy-scanning XMCD. The method was used to detect the weak XMCD signal at the Fe *K*-edge in ferrimagnetic erbium iron garnet (ErIG; Er_3_Fe_5_O_12_). The quality of the spectra and of the field dependence of the XMCD amplitude allow for a quantitative evaluation of the Fe *K*-edge signal amplitude for the study of the Fe sublattice magnetization.

## 2. Experimental set-up

**Figure 1:**
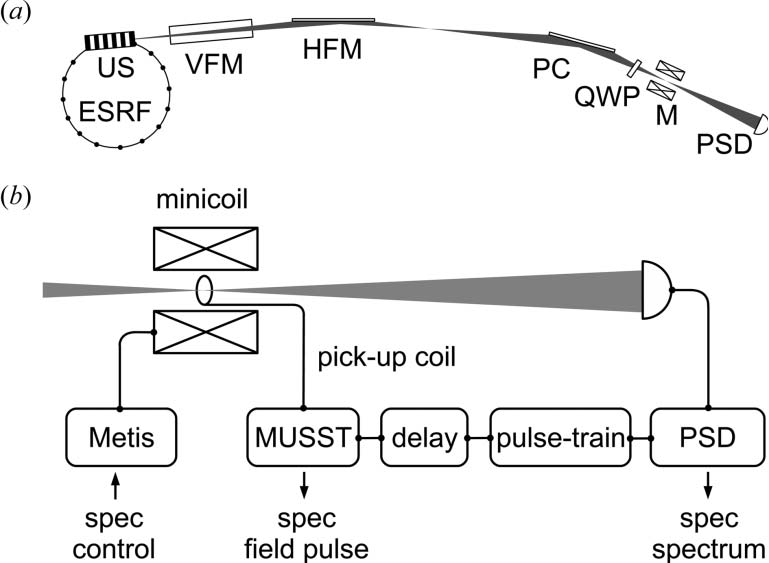
(*a*) Beamline layout (not to scale). ESRF: storage ring; US: undulator source; VFM: vertical focusing mirror; HFM: horizontal focusing mirror; PC: polychromator; QWP: quarter-wave plate; M: minicoil magnet system; PSD: position-sensitive detector. (*b*) Electronics and triggering. Metis: thyristor-switched capacitive storage pulsed power supply; minicoil: miniature pulsed magnet system; MUSST: fast data acquisition card; delay: Stanford DG535 delay generator; pulse-train: Berkeley Nucleonics Corporation BNC 555 pulse generator; PSD: Quantum Detectors ‘Ultra’ Si strip detector; spec: beamline control software.

The experiments were performed at the energy-dispersive X-ray absorption spectroscopy beamline ID24 (Pascarelli *et al.*, 2006 ▶) at the European Synchrotron Radiation Facility. Fig. 1(*a*) ▶ shows the beamline layout.

The beam from an undulator source is focused vertically onto the detector by a first mirror (VFM), which also acts as a filter to reject higher harmonics and to reduce the heat load on the optics further downstream. A second mirror (HFM) focuses the beam horizontally, creating the divergence necessary to illuminate the polychromator and to obtain the desired spectral bandwidth. An ideally elliptically bent Si (111) polychromator crystal (PC) then refocuses the beam horizontally on the sample while introducing a correlation between the propagation direction and the photon energy. Finally, a position-sensitive detector (PSD) makes use of the energy–direction correlation to simultaneously acquire the energy dependence of the transmitted intensity. In order to generate light of circular polarization for the XMCD measurements over a large bandwidth a diamond (111) quarter-wave plate (QWP) is used in a quasi non-dispersive setting with the polychromator (Pizzini *et al.*, 1998 ▶). The miniature pulsed magnet system (M) consists of a liquid-nitrogen-cooled coil reaching fields of 30 T and an independent He sample cryostat of the flow type covering sample temperatures from 5 K to 250 K. The coil is wound from Cu:Ag wire in two concentric parts with a gap for internal cooling in order to increase the pulse repetition rate. The magnet is energized through a bipolar thyristor-switched capacitive storage pulsed power supply, commercially available from Metis (http://www.metis.be/). A detailed description of the miniature pulsed magnet system is found by van der Linden *et al.* (2008 ▶).

For the PSD we used the ‘Quantum Detectors Ultra System’ Si-strip detector, developed by the STFC (Science and Technology Facilities Council, http://www.stfc.ac.uk/) and commercially available through Quantum Detectors (http://www.quantumdetectors.com/). The detector consists of a 300 µm-thick back-illuminated Si sensor with a linear array of 512 strips of pitch 50 µm and a usable strip length of 1.7 mm. The absorption at the Fe *K*-edge at 7.112 keV is about 99.8%. The detector strips are wire bonded directly to four X2CHIP readout application-specific integrated circuits. The X2CHIP contains 128 integrating amplifiers, each with four storage capacitors. The amplifier voltages are sampled at the beginning and end of the integration period on pairs of capacitors, and the voltages are then multiplexed onto the analogue outputs. Multiplexing is controlled by shift-registers and is configured as four separate 32-channel blocks. During the read-out cycle the amplifier voltages are stored on different capacitors so that readout and integration periods can be overlapped. The back-end data electronics removes common mode noise artifacts by taking a difference measurement between the X2CHIP outputs for the voltage sampled at the beginning and end of integration. This difference is then digitized by 16-bit 1 MHz analog-to-digital converters (ADCs). The output data from the ADCs are fed into a field-programmable gate array which handles formatting of the data and transfer to a host by a 1 Gbit Ethernet connection. Our version of the detector is software limited (for compatibility with existing beamline systems) to recording a sequence of a maximum of 1500 frames. The detector can be set with exposure times ranging from 2 µs to 65 µs, has a fixed readout time of 50 µs per frame and a maximum repetition rate of 20 kHz. For integration with other beamline hardware the detector integration/readout is operated in an external trigger configuration with a trigger latency of 40 ns and a trigger jitter of ±5 ns. However, the system can also be placed into a self-triggered mode. After completion of each sequence the data are transferred to a device server where they are recovered by the beamline control software.

Samples were small single crystals of ErIG oriented by Laue backscattering and mechanically polished with the surfaces perpendicular to the crystallographic (100) direction to a thickness of about 28 µm, resulting in an absorption step of 2.0 at the Fe *K*-edge. The samples were sandwiched between two 250 µm-thick Si plates with holes of diameter 500 µm, resulting in a free-standing sample in the area illuminated by the beam.

## 3. Multi-frame acquisition

Fig. 1(*b*) ▶ shows the electronics used to control the experiment and the trigger chain for the detector.

The charging and triggering of the power supply (Metis) are carried out *via* the beamline control software, as detailed by Mathon *et al.* (2007 ▶). The signal from the pick-up coil is recorded by an internally developed fast data acquisition card (MUSST), and serves to obtain the temporal profile of the field pulse through integration of the pick-up voltage (Fig. 1*a* ▶), as well as to generate a trigger for the delay and frame generators to synchronize the PSD to the field pulse. The trigger signal is passed through a delay generator (Stanford DG535) and then starts a pulse train generated by a Berkeley Nucleonics Corporation BNC 555 pulse generator to trigger the detector for the desired number of frames.

In the present experiment the XMCD signal was acquired at fixed left- or right-circular polarization while flipping the magnetic field. Each single acquisition results in an (*n* × *m*) array of intensities, where *n* and *m* are the frame and pixel indices, respectively. A typical XMCD acquisition requires about 50 to 350 pairs of positive and negative magnetic field pulses. In order to obtain the XMCD spectra from the arrays of raw data, the series of vectors corresponding to each frame index was then processed using the scheme outlined by Mathon *et al.* (2004 ▶), which eliminates the need to record (the energy dependence of) the incident intensity.

**Figure 2:**
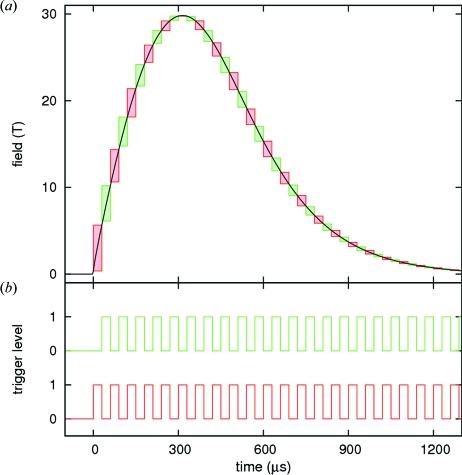
(*a*) Line: typical magnetic field pulse; boxes: acquisition windows. (*b*) Trigger sequences (schematic) for the acquisition windows shown in (*a*). The two traces were offset for clarity. Red boxes and line: delay 0 µs; green boxes and line: delay 30 µs. The period of both trigger sequences is 60 µs. The combination of acquisition series with trigger sequences shifted by half a period allows the limitation of the maximum frame rate of the detector system to be overcome.

For cases in which the maximum detector frame rate of 20 kHz is too slow to precisely follow the field dependence of the XMCD signal, such as for example for the study of phase transitions, two (*x*) acquisition series with pulse trains shifted by half (1/*x*) of the period can be acquired separately and then interleaved to increase the number of points in a field dependence. Here, two pulse trains with a period of 60 µs and an exposure time of 30 µs were interleaved (Fig. 2*b* ▶), resulting in the acquisition windows shown in Fig. 2(*a*) ▶ as red and green boxes. The period and exposure time were chosen as a compromise between the statistics per frame and the desired resolution.

**Figure 3:**
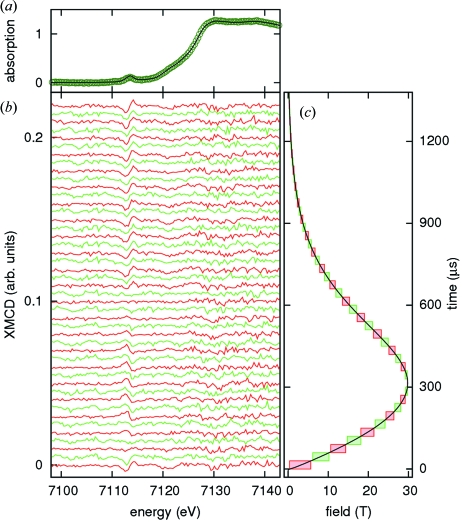
XMCD at the Fe *K*-edge in Er_3_Fe_5_O_12_ at 65 K. (*a*) Normalized absorption. Points: data taken at ID24; line: reference spectrum for energy calibration recorded at BM29. (*b*) XMCD spectra. The spectra are offset by 0.005 for clarity. (*c*) Field pulse and acquisition windows corresponding to the spectra in (*b*). Red boxes and lines: delay 0 µs; green boxes and lines: delay 30 µs.

Fig. 3(*a*) ▶ shows the normalized absorption in ErIG at 65 K taken at the beamline ID24 (points) along with an ambient temperature spectrum from a powder sample recorded at beamline BM29 (line) that was used to calibrate the energy scale of the PSD. Fig. 3(*b*) ▶ shows the XMCD spectra obtained in two series of 350 pairs of positive and negative field pulses for each set of acquisitions. The whole energy range is affected by a floor of spatial noise, whereas the difference in the noise level below and above the absorption edge is mainly due to photon count statistics. The flux after the sample was estimated to be 4 × 10^8^ and 5 × 10^7^ photons stripe^−1^ s^−1^ for energies below and above the edge, respectively (note that the profile of the incident flux is not flat over the energy spectrum). Fig. 3(*c*) ▶ once more shows the corresponding acquisition windows for reference. In the present experiment the miniature coil was operated with a repetition rate of approximately 2 pulses min^−1^ and the entire data set was acquired in just under 12 h.

## 4. Results

The experiments were carried out on samples of ErIG which crystallizes in the cubic garnet structure. The cations are located at the centers of oxygen polyhedra with different symmetries. Per formula unit, two Fe occupy sites of octa­hedral and three Fe occupy sites of tetrahedral symmetry, whereas three Er are in a dodecahedral environment. Each site further occurs in different orientations within the unit cell. The cations interact magnetically *via* an antiferromagnetic exchange over the oxygen anions, where the relative strength of the interaction is determined mainly by the bond angles and distances. The antiferromagnetic interaction between the tetrahedral and octahedral Fe sites is strongest and remains unaffected up to high temperatures and very high applied fields. The interactions between the Fe sites and the Er sites are, however, much weaker and the fields available in the present experiment are comparable with the corresponding molecular field. In this context the system can thus be approximately described as a two-sublattice ferrimagnet (Clark & Callen, 1968 ▶) between the net Fe and the Er moments. At high temperatures the Fe net magnetization dominates, and aligns with an applied field. With decreasing temperature the magnitude of the Er magnetization increases and both the Fe and Er sublattices reverse orientation with respect to the applied (low) field at the compensation temperature *T*_comp_. Below *T*_comp_ the system undergoes two successive phase transitions as a function of the applied field (Nakao *et al.*, 1986 ▶). Below *H*_l_ the Er sublattice is aligned with the field while the net Fe moment is opposite; between *H*_l_ and *H*_u_ the Er and Fe moments form a canted phase in which the net Fe magnetization undergoes a continuous reversal while the Er spins are canted away from the applied field; and above *H*_u_ both sublattices become aligned with the external field.

**Figure 4:**
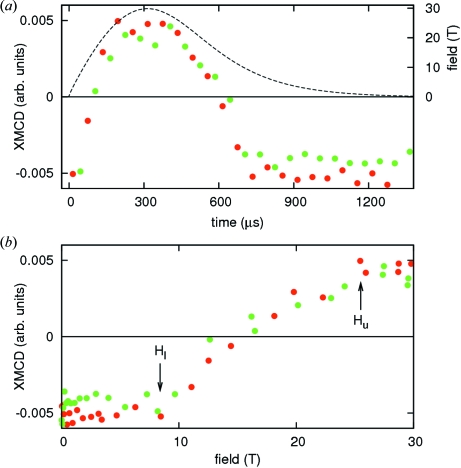
XMCD at the Fe *K*-edge in Er_3_Fe_5_O_12_ at 65 K. (*a*) Evolution of the XMCD amplitude (points) during the field pulse (dashed line). (*b*) Field dependence of the XMCD amplitude. Red and green color denote data taken in acquisition sequences with delays of 0 µs and 30 µs, respectively.

In the present experiments the beam and the magnetic field were parallel to the crystallographic (100) direction. The sample was cooled below the compensation point (*T*_comp_ ≃ 79 K) at a temperature of 65 K. The XMCD signal visible in Fig. 3 ▶ coincides with the pre-peak which is attributed to the sites of tetrahedral symmetry (Kawamura *et al.*, 1997 ▶; Maruyama & Kawamura, 2004 ▶). For the interpretation we here make the simplifying assumption that the pre-edge signal is directly related to the tetrahedral Fe sites and thus to the net magnetization of Fe sublattices. At the main edge there is barely any XMCD signal visible with the available resolution. In the raw spectra in Fig. 3 ▶ a decrease, disappearance, reversal of sign and subsequent increase of the magnitude of the XMCD signal as a function of field are clearly observable. In order to plot the field dependence of the XMCD signal its amplitude was evaluated as the difference between the averages over the strips 174–176 and 178–180 in the raw spectra, which correspond to the minima and maxima centered at 7112.9 eV and 7114.2 eV, respectively. Fig. 4(*a*) ▶ shows the evolution of this amplitude during the magnetic field pulse, and Fig. 4(*b*) ▶ shows the resulting field dependence.

Below ∼8.4 T the amplitude barely evolves with the field; at ∼8.4 T the amplitude starts to decrease, crosses zero and continues to decrease until it saturates around ∼25.5 T at a magnitude slightly lower than the low field value, and opposite in sign. The data clearly reflect the expected behavior for the Fe sublattice magnetization and the transition fields are in agreement with the phase diagram published by Nakao *et al.* (1986 ▶). A more detailed description and interpretation of the scientific results will be given elsewhere (Strohm *et al.*, 2011 ▶).

## 5. Conclusion and outlook

We have implemented a multi-frame acquisition scheme to perform ED-XMCD in pulsed high magnetic fields. This detection scheme allows the entire field pulse to be followed and thus combines the high quality of the energy spectra obtained in the dispersive geometry with a good consistency of the field dependence. The multi-frame detection significantly increases the overall efficiency of energy-dispersive experiments in pulsed magnetic fields. At the moment the speed of the available detector system is limited to 20 kHz. It was shown that higher temporal resolution can nevertheless be obtained by interleaving several acquisition series when required. The multi-frame acquisition scheme was used to perform Fe *K*-edge XMCD in samples of ErIG. Up to now, most, if not all, XMCD experiments in pulsed fields have been performed at *L*-edges, where the signal is typically of the order of several percent. The possibility to quantitatively exploit the small Fe *K*-edge signal significantly enlarges the number of accessible subjects. In the future we expect to use other *K*-edges in the energy range compatible with ID24 and the Ultra System, in particular the *K*-edges of Mn, Co, Ni and Cu. For the *L*-edges in the accessible energy range we may hope to be able to detect even small induced moments. We have studied the field dependence of the Fe *K*-edge XMCD signal in ErIG, below the compensation point. The pre-peak signal, associated with the Fe sites of tetrahedral symmetry, allows two successive phase transitions to be identified and the reversal of the net magnetization of the Fe sublattices in the intermediate canted phase to be observed.
